# On Theoretical and Numerical Aspects of Bifurcations and Hysteresis Effects in Kinetic Energy Harvesters

**DOI:** 10.3390/s22010381

**Published:** 2022-01-05

**Authors:** Grzegorz Litak, Jerzy Margielewicz, Damian Gąska, Andrzej Rysak, Carlo Trigona

**Affiliations:** 1 Faculty of Mechanical Engineering, Lublin University of Technology, 20-618 Lublin, Poland; a.rysak@pollub.pl; 2 Faculty of Transport and Aviation Engineering, Silesian University of Technology, 40-019 Katowice, Poland; jerzy.margielewicz@polsl.pl (J.M.); damian.gaska@polsl.pl (D.G.); 3 Dipartimento di Ingegneria Elettrica Elettronica e Informatica, University of Catania, 95125 Catania, Italy; carlo.trigona@dieei.unict.it

**Keywords:** vibration energy-harvesting system, hysteretic effect, bistable oscillator, bifurcation

## Abstract

The piezoelectric energy-harvesting system with double-well characteristics and hysteresis in the restoring force is studied. The proposed system consists of a bistable oscillator based on a cantilever beam structure. The elastic force potential is modified by magnets. The hysteresis is an additional effect of the composite beam considered in this system, and it effects the modal solution with specific mass distribution. Consequently, the modal response is a compromise between two overlapping, competing shapes. The simulation results show evolution in the single potential well solution, and bifurcations into double-well solutions with the hysteretic effect. The maximal Lyapunov exponent indicated the appearance of chaotic solutions. Inclusion of the shape branch overlap parameter reduces the distance between the external potential barriers and leads to a large-amplitude solution and simultaneously higher voltage output with smaller excitation force. The overlap parameter works in the other direction: the larger the overlap value, the smaller the voltage output. Presumably, the successful jump though the potential barrier is accompanied by an additional switch between the corresponding shapes.

## 1. Introduction

Mechanical vibrations typically induced during machine operation are a disadvantageous phenomenon. In most practical applications, the influence of mechanical vibrations is limited by means of vibration-reduction systems, while the complete elimination of vibration is practically impossible. On the other hand, there are devices in technology in which vibrations are intentionally induced: vibrating conveyors, compactors, pneumatic hammers, etc. Currently, there is growing interest from both scientists and industry in the harvesting of this irretrievably lost energy resulting from vibrations [1,2,3]. This is possible because of energy harvesters that use, among others, the piezoelectric effect.

Piezoelectric energy harvesting from ambient vibration sources has been widely studied in recent years [1,2,3,4,5,6,7,8,9,10,11]. The applied devices are made up of a vibration resonator formed as a beam-mass resonator structure and a piezoelectric transducer [1,2]. To improve efficiency, a nonlinear oscillator was proposed [3,4,5,6,7,8,9,10,11,12], which is characterized by inclinations of resonance curves [13] and additional resonances defined by the multiplied rational and fractions of the main resonance frequencies [11,12,14]. Simultaneously, multiple coexisting solutions are present in such a system and depend on initial conditions [13,14,15]. Among the main disadvantages of such systems, one can distinguish the difficulty of controlling particular solutions [16]. In recent years, several studies have been conducted to investigate, among others, the hysteretic effects of elastic beam materials and related structures, as well as piezo elements [17,18,19]. In this study, we continue the investigation of the dynamics of a nonlinear bistable energy harvester with hysteresis induced by the snap-through phenomenon of a bistable beam [17,19]. Our study was inspired by a recent work [17], wherein composite bistable beams were used for energy harvesting, and by [20], where bistability was used in combination with an additional magnetic field to model the potential shape. In [17], the authors proposed a piezoelectric energy harvester that stores the potential energy induced by the mutual self-constraint of the subbeams and harvests the large energy released during the rapid shape transition.

Regarding nonlinear systems, the assessment of the impact of individual parameters is difficult to implement without detailed numerical experiments [8,12]. This claim is justified because even a small change in the value of any parameter in a nonlinear system can lead to drastic changes in the dynamics of the system [21]. In this publication, we are considering cutting the potential barrier and shifting the cut parts so that they overlap. This can be modelled by an elastic cantilever beam that is subjected to initial elastic deformation. An example of such an arrangement would be a typical hairpin. Another possibility is to use a shape-memory material.

## 2. Mathematical Model

**Figure 1:**
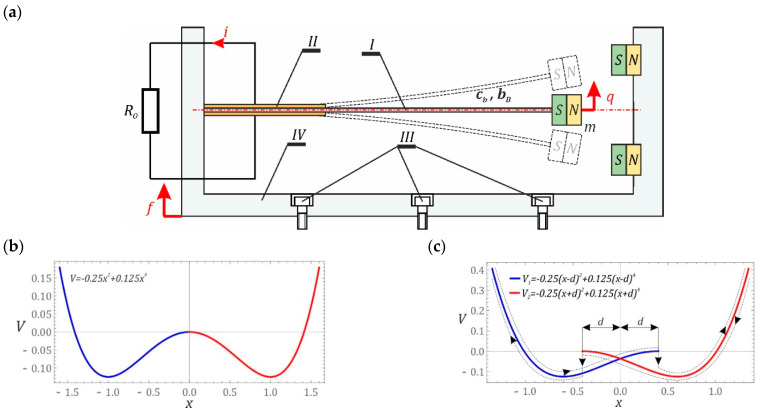
System and its potential characteristics: (**a**) schematic diagram without indicating the origin of hysteresis, modelling of the hysteretic branches: (**b**) potential without hysteresis loop, (**c**) with hysteresis loop, *d* indicates the overlap shift, *q* is the displacement of the tip point of the beam. *i* is the current in the electrical circuit.

The starting point for the numerical experiments performed is the piezoelectric beam resonator system (Figure 1a) [22], which is the subject of many publications (see [23]). In general, the analyzed system consists of a flexible beam I with piezoelectric transducers II attached to its flat surfaces. The flexible beam I is rigidly fixed in the frame IV, which is bolted to the vibrating subassembly of the mechanical system, from which energy is recovered by means of screws III. In our research, the impact of the hysteresis loop (presumably from the composite beam) on the efficiency of the energy harvested was assessed. For simplicity, we used the initial configuration of the system without hysteresis (Figure 1a) accompanied by the interacting permanent magnets to support the double-well solution (Figure 1b). The hysteresis loop caused by the beam complexity is introduced to the above system potential as an effect of cut and sift the left- and right-hand sides of potential characteristic (Figure 1c) with respect to the central point at the top of the potential barrier by the distance of d (Figure 1c).

In numerical simulations, it was assumed that the system was influenced by mechanical vibrations of the frame mapped by the harmonic function having an amplitude A and a frequency *ω_W_*, *f* = *A*sin(*ω_W_ t*). The differential equations of motion, taking into account the electromechanical coupling, have a general form of:

(1) $$\{\begin{array}{l} m_{1}\frac{d^{2}q}{dt^{2}}+b_{B}\left(\frac{dq}{dt}-\frac{df}{dt}\right)+c_{B}\left(q-f\right)-c_{1}\left(q-f\right)+c_{2}{\left(q-f\right)}^{3}+k_{P}U_{P}=0,\\ C_{P}\frac{dU_{P}}{dt}+\frac{U_{P}}{R_{O}}-k_{P}\left(\frac{dq}{dt}-\frac{df}{dt}\right)=0, \end{array}$$

where *m*_1_, *b_B_*, *c_B_* are effective (modal) beam parameters corresponding to the mass, damping and stiffness, respectively. *c*_1_ and *c*_2_ denote the coefficients of the magnetic force. *C_P_* and *R_O_* are parameters in the electrical circuit indicating capacity and resistance, respectively. Finally, *k_p_* is the coupling parameter and *U_p_* is the voltage output on the resistor [4,12,22].

Considering quantitative and qualitative computer simulations, the system of Equation (1) was transformed into a dimensionless form. A new coordinate *y*, *y* = *q* − *f*, was introduced, defining the difference between the displacement of the free end of the flexible beam *q* and the point of its attachment to the rigid frame IV *f* (see Figure 1a). The differential equations of motion, considering dimensionless time and dimensionless displacement, finally take the form:

(2) $$\{\begin{array}{l} \ddot{x}+2\delta \dot{x}-\alpha \left(x+a\cdot d\right)\cdot \left[1-{\left(x+a\cdot d\right)}^{2}\right]+\theta u=\omega^{2}p\sin\left(\omega \tau \right),\\ \dot{u}+\sigma u-\vartheta \dot{x}=0. \end{array}$$

where:

$$\begin{array}{c} \begin{array}{ccccc} \omega_{0}^{2}=\frac{c_{1}-c_{B}}{m}, & \delta =\frac{b_{B}}{m\omega_{0}}, & \alpha =\frac{c_{2}x_{0}^{4}}{c_{1}-c_{B}}, & \theta =\frac{k_{P}}{\omega_{0}^{2}x_{0}m}, & p=\frac{A}{x_{0}}, \end{array}\\ \begin{array}{ccccc} \omega =\frac{\omega_{W}}{\omega_{0}}, & x=\frac{y}{x_{0}}, & \vartheta =\frac{k_{P}x_{0}}{C_{P}}, & \sigma =\frac{1}{\omega_{0}C_{P}R_{O}}, & \tau =\omega_{0}t. \end{array} \end{array}$$

In the model tests, *x*_0_ represents the scaling parameter equal to the absolute value of the coordinate defining the position of the minimum potential barrier. On the other hand, the variable a occurring in the mathematical model reflects control quantity responsible for the shift of the operating point from one half of the potential barrier to the other. In general terms, the variable a takes the value of 1 or −1 depending on the hysteretic branch. Here, we limit ourselves to listing the numerical values of the mathematical model coefficients, based on which quantitative and qualitative computer simulations were carried out, i.e.,: *δ* = 0.05, *ϑ* = 0.5, *θ* = 0.05, *σ* = 0.05, *α* = 0.5.

## 3. The Results of Model Tests

**Figure 2:**
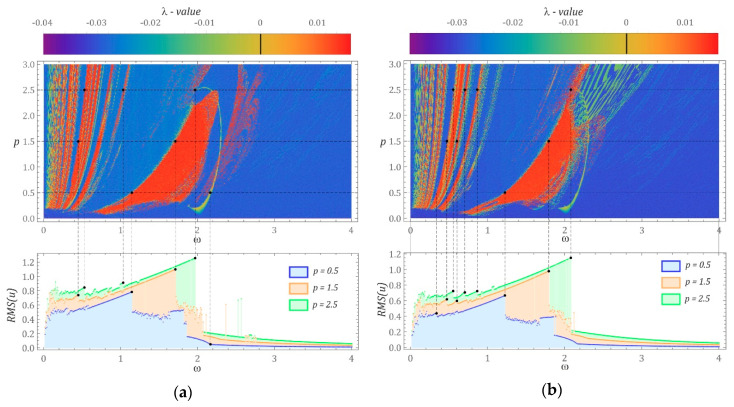
Influence of the parameter *d* and damping on the distribution of the largest Lyapunov exponent: (**a**) *d* = 0, (**b**) *d* = 0.3 with nodal initial conditions.

Based on the assumed numerical data characterizing the mathematical model of the analyzed energy-harvesting system, numerical experiments were performed to investigate the impact of the d shift on the efficiency of energy harvesting. In the first stage, the impact of the shift on the location of the zones of chaotic and periodic solutions was assessed. Among the periodic solutions, small and large orbits were found. Areas of chaotic solutions can be identified via different numerical tools, such as bifurcation diagrams [21], 0–1 test [19,24] or by determining the maximal Lyapunov exponent [25]. In our numerical simulations (performed in Mathematica), the areas of chaotic and periodic solutions were visualized in the form of multicolor maps showing the distribution of the maximal Lyapunov exponent (Figure 2). According to the authors, this approach makes it possible to look at system dynamics with a wide range of variability of the parameters characterizing the source of mechanical vibration affecting the energy-harvesting system. The maximal Lyapunov exponent was estimated in a three-dimensional phase space following the numerical procedure proposed by Wolf et al. [26]. The phase space was spanned by the computed coordinates (*x*, $\dot{x}$, *u*). All the included two-dimensional, multicolor distribution maps were plotted for the assumed nodal initial conditions: *x*(0) = 0, $\dot{x}$ (0) = 0 and *u*(0) = 0. Furthermore, two control parameters, ω and p, were selected to describe changes in the external excitation source. Technically, the small distance of the initial conditions between the tested trajectory and the reference trajectory was assumed to be ε(0) = 10^−5^. To obtain a satisfactory resolution, the ranges of the control parameters ω and *p* were divided into 500 subintervals.

It is worth mentioning that the positive values of λ relate to the chaotic dynamic response of the system, otherwise (negative, λ < 0) the system response is regular with the corresponding phase trajectories tending to stable points or periodic orbits. However, when λ approaches values equal to zero, we are dealing with the so-called bifurcation points (or quasiperiodic solutions). In a low frequency range ω, narrow repetitive zones of chaotic solutions are arranged along the curves that can be approximated by functions with a fairly high exponential growth. Regardless of the parameter *d* value, the largest area of chaotic solutions is located in the central parts of the multicolor maps showing the distribution of the maximal Lyapunov exponent. For the case of *d* = 0, this region is located in the band ω [1,2] (see Figure 2a). However, for *d* = 0.3, this zone stretches towards higher values of the dimensionless frequency ω [1, 2.4] (see Figure 2b). An indicator expressed as the effective value of the voltage induced on the piezoelectric electrodes was taken as a measure of energy-harvesting efficiency. To determine the effect of the solution on the efficiency of energy harvesting, the multicolor maps of the maximal Lyapunov exponent distribution were compared with the diagrams of effective voltage values. The results clearly indicate that when the solution changes from periodic to chaotic, the voltage induced on the piezoelectric electrodes is limited. Examples of these landmarks are marked in black. The RMS(*u*) diagrams (RMS—root mean square) were identified in relation to every value of the dimensionless excitation frequency from the range ω [0,4], based on a time sequence of 50 periods of the mechanical vibration signal affecting the energy-harvesting system.

With regard to the zero initial conditions, it can be observed that—irrespective of the displacement value of the “cut” halves of the potential barrier d (Figure 1b,c) and the amplitude of the excitation source p—periodic solutions with a periodicity of 1T (single excitation period T) dominate in the range of low, dimensionless excitation frequencies of *ω* < 0.5. It should be noted that increasing the width of the hysteresis loop reduces the energy-harvesting capability. Consequently, the reduction in the voltage induced on the piezoelectric electrodes is particularly noticeable in the range of higher parameter *p* values. In the range of low amplitudes of forced vibration, *p* < 0.1, particularly in the zone of chaotic solutions, no significant decrease in effective voltage value is observed. As predicted by RMS(*u*), the voltage value is directly proportional to the amplitude and average kinetic energy of cantilever beam vibration.

### 3.1. Influence of the Parameter d on the Geometrical Structure of a Chaotic Attractor

**Figure 3:**
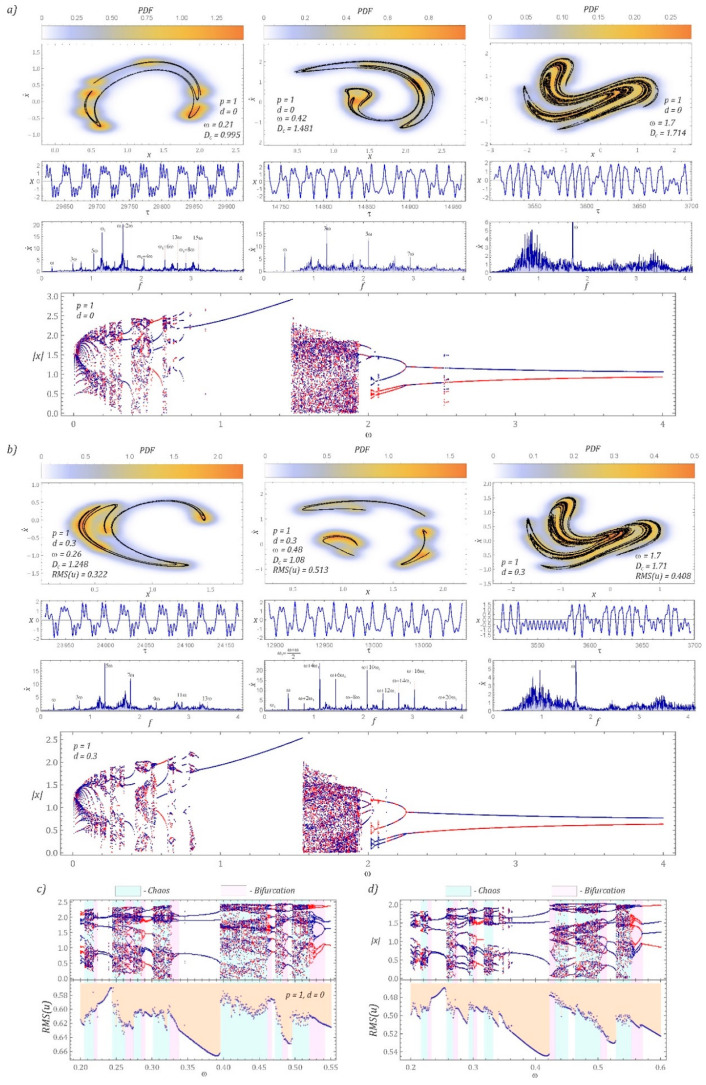
Influence of the parameter *d* on the geometric structure of the Poincaré cross-section: (**a**) *d* = 0, (**b**) *d* = 0.3. Starting from the top, the horizontal panels correspond to the Poincaré map and the corresponding time series for selected ω and amplitude–frequency spectrum. The bottom panels show the bifurcation diagrams based on the local minima (red) and the local maxima (blue) compared with RMS(u) for *d* = 0.3. To clarify the influence of the parameter *d* on bifurcations in the selected frequency interval, (**c**,**d**) magnify the difference between the cases *d* = 0 and *d* = 0.3, respectively. The simulation results were obtained assuming zero initial conditions.

The below graphs (Figure 3) show examples of attractors that occur in the regions of chaotic solutions. It is worth mentioning that in the classic visualization of the Poincaré cross-section, numerical results are presented as a set of points located on the phase plane. Much more information about the geometric structure of chaotic attractors can be obtained by analyzing the density of the distribution of the points of intersection of the trajectory and the control plane. In this way, one obtains information from the areas of the phase plane where the trajectory most often intersects with the control plane. The graphical representations of the Poincaré cross-sections are then plotted against bifurcation diagrams. From a theoretical point of view, bifurcation diagrams can be drawn based on the following: Poincaré cross-section, phase trajectory and time sequence. In our case, these diagrams were plotted based on the local minima (points marked in red) and maxima (points marked in blue) of the analyzed time series (Figure 3).

The plotted Poincaré cross-sections show different geometrical structures of attractors depending on the external excitation frequency value affecting the energy-harvesting system. To quantify the geometric structure of the chaotic attractors, their correlation dimension was identified, and the effective voltage values induced on the piezoelectric electrodes were also given. Based on the model tests, it was found that in the case of the “developed” attractors (a developed attractor is understood as a structure consisting of the Poincaré cross-section points forming an even distribution along the geometric structure), the correlation dimension of the attractor of the system with a displaced characteristic (*ω* = 1.7 Figure 3b) is comparable to the attractor plotted for the classical potential function (*ω* = 1.7 Figure 3a). In conclusion, the parameter *d* does not significantly affect the position of the band of chaotic solutions. Both for *d* = 0 and *d* = 0.3, unpredictable solutions are located in comparable ranges of the variability *ω* ϵ [1.5, 1.9]. Similar values of the correlation dimension (DC) also result from the similarity of the geometric structure of the chaotic attractors and the density distribution of the points of intersection between the trajectory and the control plane.

The most important element distinguishing the two discussed cases is the parameter representing the effective value of the voltage induced on the piezoelectric electrodes. In the case of the “split” and shifted characteristic (*ω* = 1.7 in Figure 3b), the value is lower by approximately 0.08. This RMS voltage value is directly related to the lower amplitude of the displacement of the free end of the flexible beam I (Figure 1). The influence of the displacement of the “cut” halves of the potential barrier is best visible in the bifurcation diagrams. In particular, this is very visible in the range of low values of *ω*, where increasing the parameter *d* results in narrowing the zones of chaotic solutions. It is also worth noting that in the range of low values of ω, the zones of unpredictable solutions are additionally shifted, as a result of which it is impossible to directly refer to the Poincaré cross-sections that were plotted for the cases *d* = 0 and *d* = 0.3. The numerical modelling results presented in this section were obtained for zero initial conditions. This approach however does not fully reflect the capability of harvesting energy from vibrating mechanical devices. Therefore, the numerical simulations focused on the assessment of initial conditions from the point of view of coexisting solutions.

With reference to the presented Poincaré sections, Fourier amplitude–frequency spectra were plotted. They provide information on the dominant harmonics in the time series, which constitutes the formal basis for depicting Poincaré maps. Based on the spectra, it is possible to conclude that in the case of developed attractors, for which the correlation dimension *D_C_* > 1.5, harmonic components representing the frequency of the source of excitation dominate in the Fourier spectra. We deal with such geometrical structures of Poincaré cross-sections in the widest zone of chaotic solutions, located in the band *ω* ϵ [1.4, 1.9]. However, in the range of low ω values, the correlation dimension of the Poincaré cross-sections *D_C_* <1.5. At the same time, in the amplitude–frequency spectra, it is represented by the domination of harmonic components that are a multiple of the frequency of mechanical vibrations affecting the energy-harvesting system. We deal with such a sequence of dominant harmonic components of the Fourier spectrum when the correlation dimension of Poincaré cross-sections is in the *D_C_* range of [1.1, 1.5]. At this point, it is worth noting that such spectra occur both in the case of continuous and smooth (*p* = 1, *d* = 0, ω = 0.42) and discontinuous (*p* = 1, *d* = 0.3, *ω* = 0.26) characteristics representing the potential barrier. On the basis of the presented results of numerical experiments, it is also possible to state that for correlation dimensions *D_C_* < 1.1, in the amplitude–frequency spectra, the dominant harmonic components are the multiples of the combination of the two fundamental frequencies ω and ω1. As in the previous example, this is the case with both continuous and smooth (*p* = 1, *d* = 0, *ω* = 0.21) and discontinuous (*p* = 1, *d* = 0.3, *ω* = 0.48) characteristics representing the barrier potentials.

The diagrams (the bottom panels Figure 3a,b) show a direct comparison of Feigenbaum’s steady state bifurcation diagrams with the diagrams of RMS values of the voltage induced on the piezoelectric electrodes. In the wide range of variability of the dimensionless excitation frequency *ω* ϵ [0, 4], bifurcation diagrams are dominated by one relatively wide band of chaotic solutions, within the *ω* range of [1.4, 1.9]. On the other hand, in the band *ω* < 1, there are many bands corresponding to unpredictable solutions, separated by pieriodic areas and bifurcation zones. The individual bands characterizing the dynamics of the tested energy-harvesting system were distinguished by colors: the bands of chaotic solutions were depicted with a light cyan color, and the bifurcation zones with a light magenta color. Based on the presented graphs, it is difficult to unambiguously characterize the dynamics of the system, because both in the bands of chaotic solutions and bifurcation zones we deal with an increase and a decrease in the effective voltage induced on piezoelectric electrodes. We are also dealing with both the decrease and increase in the voltage induced on piezoelectric electrodes in the areas of periodic solutions. For example, the voltage drop induced on piezoelectric electrodes in the area of periodic solutions is in the ω band of [0.225, 0.245] (Figure 3c). The same is the case when the halves of the “cut” potential barrier overlap with *ω* in [0.23, 0.255] (Figure 3d), and the range of variation of the dimensionless excitation frequency is similar. In the remaining bands of periodic solutions, for example *ω* ϵ [0.337, 0.395] (Figure 3c) and *ω* ϵ [0.355, 0.42] (Figure 3d), we deal with the opposite situation, i.e., an increase in the voltage induced on the electrodes is observed. While any detailed inference regarding the correlation of the bifurcation diagram with the diagram of rms voltage values in the bands of chaotic solutions and bifurcation zones is difficult due to the large number of points, in the case of periodic solutions it is possible to formulate a probable diagnosis.

It is possible to formulate the hypothesis that if the mean of the slope coefficients approximating the branches of the bifurcation diagram in the area of periodic solutions is positive, then we are dealing with an increase in the effective value of the voltage induced on the piezoelectric electrodes. On the other hand, when the average value of the slope approximating branches of bifurcation diagrams takes negative values, a decrease in the RMS voltage is observed on the electrodes. If the mean value of the slope of the branch takes values in the vicinity of zero, then the RMS voltage diagram assumes a constant value.

### 3.2. Identification of Multiple Solutions

**Figure 4:**
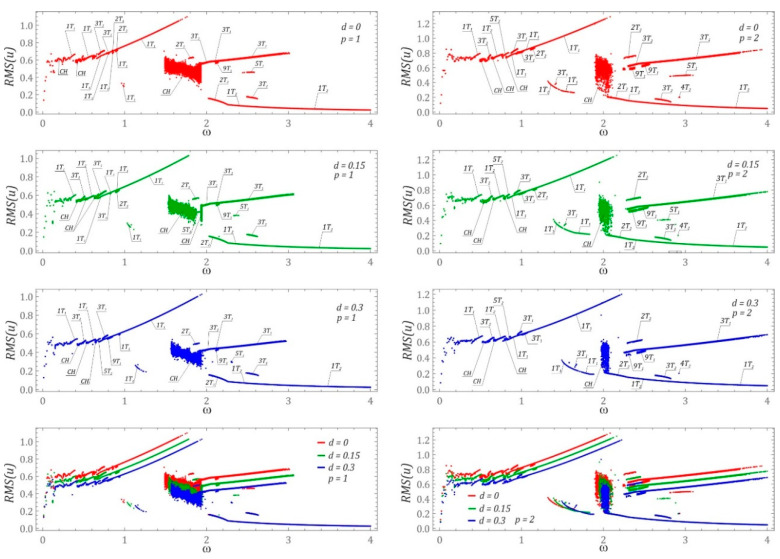
Bifurcation diagram: effect of parameters *d* and *p* on energy recovery efficiency with various initial conditions considered simultaneously for the given system parameters. The parameters are indicated in the figures. For better clarity, the solutions are marked by *nT_m_* (*n*—the response period, *m*—the number of different solutions with the same response period). The bottom panels are the summery of the upper left and right parametric cases to show the tendencies of the system evolution with *d* parameter changes.

It should be emphasized that the results presented in the previous section pertain to selected cases of system dynamics for zero initial conditions. However, one of the most important properties of nonlinear dynamic systems is related to the coexistence of different solutions depending on the initial conditions. For the defined system and excitation parameters, one has to find the system’s evolution with various initial conditions. This problem comes down to the study of phase plane orbit topologies [22], the origins of which are located in different places. In the case of systems with a greater number of degrees of freedom, coexisting periodic and chaotic solutions are identified in a multidimensional phase space. The space dimension is a multiple of the number of degrees of freedom. Considering the research problem formulated in this way, it is possible to conduct complex numerical calculations, the results of which can be illustrated in the form of a diagram of solutions (DS) [27,28]. In this approach, the information about the efficiency of harvesting energy from coexisting solutions is neglected. For this reason, this work contributes to the state of the art by proposing a different approach, the essence of which boils down to multiple plotting of voltage effective value diagrams. Every diagram is plotted for randomly chosen initial conditions of the phase space. Such presentation of computer simulation results provides information about the number of coexisting solutions. However, it does not provide information about their periodicity. For this reason, additional detailed computer simulations were performed to identify the periodicity of individual branches appearing in the diagrams of effective voltage induced on the piezoelectric electrodes (Figure 4). The following convention was adopted to define periodicity: the digit before the letter T indicates the periodicity of the solution, while the number of solutions with a given periodicity is denoted by the right subscript.

It should be noted that both the plotted DS diagrams and the diagrams of effective values plotted for randomly selected RMS(*u*) initial conditions were obtained in a simplified manner. As a result, their accuracy is a compromise between the precision of numerical calculations and the time of computer simulation. Below are some examples of diagrams showing the effective values of the voltage induced on the piezoelectric electrodes. On their basis it was possible to determine the ranges of variability of the dimensionless frequency ω in which energy harvesting is most effective. Examples of numerical results showing the effects of the dimensionless excitation amplitude p and the shift d of the left and right potential halves overlap in the barrier zone are given in the graphs (Figure 4).

Given the identified branches on the plotted diagrams of the RMS values of the voltage induced on the piezoelectric electrodes, additional numerical simulations were performed to identify phase trajectories of the coexisting solutions. The periodicity of individual branches was identified for the frequency *ω* > 0.4. Irrespective of the *d* shift value of the “cut” potential barrier, the highest energy-harvesting efficiency was obtained for 1T-periodic solutions in the band *ω* < 2. A comparison of the numerical results presented in this section with the RMS(*u*) diagrams (Figure 2) reveals that the highest energy-harvesting efficiency in this band is achieved by assuming zero initial conditions. However, in the zone *ω* > 2, energy-efficient solutions can be obtained too, for example by initiating appropriate initial conditions. It is also worth noting that the parameter *d* does not affect the nature (periodicity) of a given solution. Its influence is mainly visible in the shift of individual branches in relation to the frequency axis ω. However, with increasing the parameter *d* value, this shift is directed towards higher values of the dimensionless excitation frequency ω. Moreover, as the parameter *d* is increased, the efficiency of energy harvesting is reduced. A detailed examination of the single points and branches in the diagrams (Figure 4) shows that they represent transient solutions which finally become attracted to permanent periodic solutions over long enough time spans. This is the case with, e.g., the 4T_2_ branch that appears in the diagrams identified for *p* = 2.

**Figure 5:**
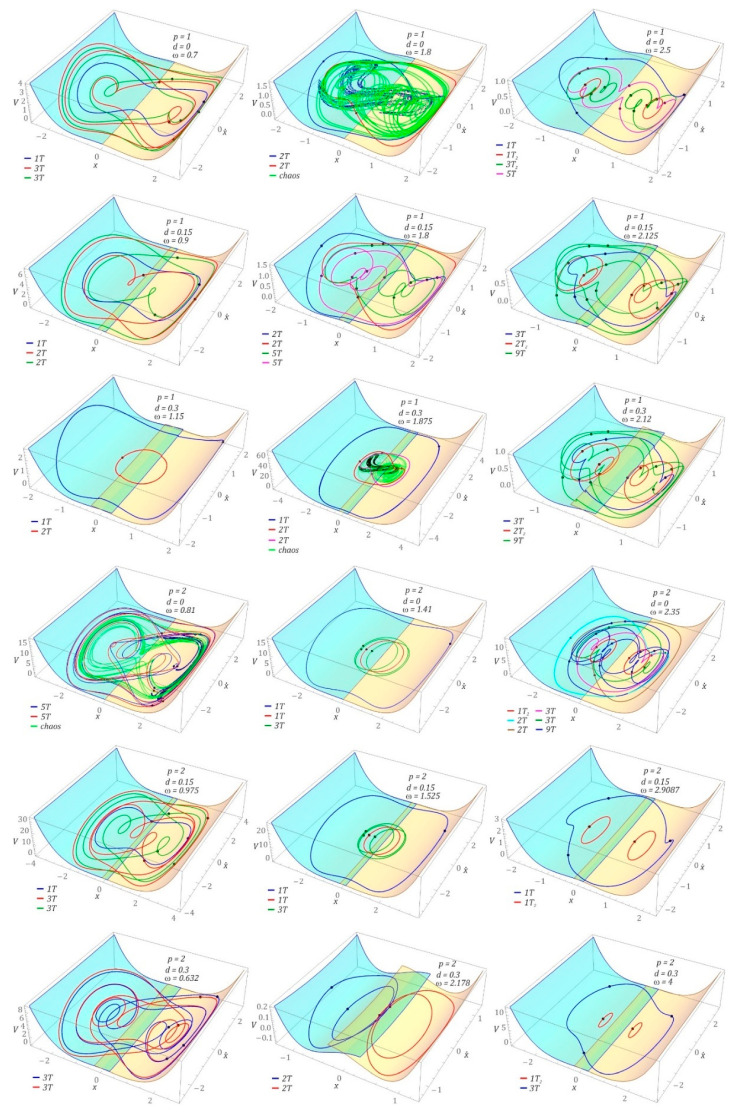
Influence of the parameters d and p on the efficiency of energy harvesting: 3D orbits with the vertical axis indicate the total mechanical energy. For clarity, the corresponding hysteretic potential is plotted with a division into two-color flaps with overlap. The system parameters are indicated in the figures.

For clarity, the selected cases were studied with the help of phase plane trajectories (Figure 5). The images are shown against the background of the potential barrier. It should be noted that the multiple solutions, which are marked in different colors depending on the solution periodicity, occur with the evolution of the frequency *ω*. The Poincaré points are also identified, which confirms the periodicity of the solutions obtained. The multiple solutions of the same period are plotted using the same color. Usually, they are trapped in the left and right potential wells.

**Figure 6:**
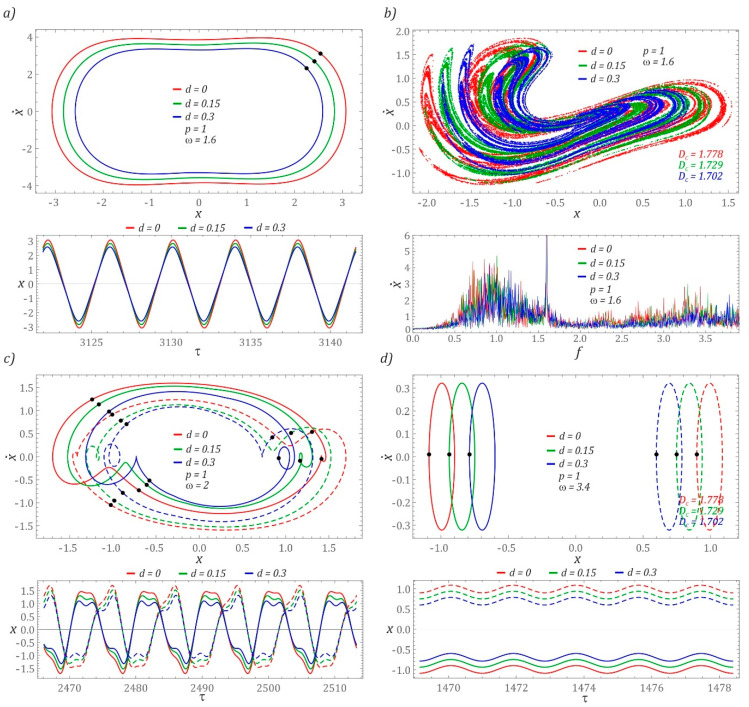
Examples showing the influence of parameter *d* on coexisting solutions, plotted for: (**a**) and (**b**) coexisting solutions for *p* = 1, *ω* = 1.6, (**c**) *p* = 1, *ω* = 2.0, (**d**) *p* = 1, ω = 3.4.

The trajectories, which were depicted against the background of the “cut” and shifted halves of the potential barrier, illustrate the possible cases of coexisting solutions. Figure 6 presents selected examples of coexisting solutions. The examples were selected to illustrate the effect of the superimposition of the “cut” halves of the potential barrier, with respect to large (Figure 6a) and small (Figure 6d) orbits of coexisting solutions and unpredictable responses (Figure 6b). The following convention was adopted during the graphical visualization of the results of computer simulations: the colors of the coexisting solutions were assigned to the individual branches of the diagrams of the RMS values of the voltage induced on the piezoelectric electrodes (Figure 4), while the coexisting solutions are plotted with dashed lines.

Based on the results of computer simulations, it can be concluded that the increase in the value of the parameter *d* does not change the nature of the solution. This behavior of the system is observed both in the case of periodic and chaotic solutions. It is also worth noting that the vibrations of the elastic cantilever beam for periodic solutions do not undergo a phase shift with the increase in the parameter *d* (Figure 6a,c). In the case of large and medium orbits, only a reduction in the amplitude of vibrations is observed. However, amplitude limitations were not observed for solutions whose orbits are located inside the potential well (Figure 6d). If the response of the system is given in the form of a chaotic solution (Figure 6b), the change of parameter *d* does not cause a significant deformation of the geometric structures of the Poincaré sections, and their similarity is preserved. The differences in the plotted Poincaré cross-sections become apparent when the correlation dimensions are identified. With an increase in the value of the parameter *d*, a decrease in the value of the correlation dimension *Dc* is observed. On the other hand, in graphical images of amplitude–frequency spectra, it is manifested by limiting the amplitude of the excited harmonics, with a simultaneous slight shift towards higher values.

## 4. Conclusions

The influence of parameter *d* reduces the distance between the external potential barriers, and as a result, the efficiency of energy harvesting changes. On the one hand, the system easier undergoes the potential barrier, on the other, the large orbit size is limited. The small orbit solutions are very similar for any d as they are governed by the linearized equations. In the hysteretic potential case, chaotic solutions may appear easier as a result of the higher competition between unstable small and large orbits. It is worth noting that the hysteretic property of the system can be a side effect of bistable structures such as bistable plates or beams [20,29]. Consequently, the amplitude of resonator oscillations is smaller, and the efficiency of energy harvesting is decreased with respect to the system without hysteresis. In the next step, we would perform experimental verification of the observed tendencies with a suitable metrological characterization. Based on the numerical experiments carried out, it is possible to formulate the following, more detailed conclusions:

- If the mean value of slope coefficients approximating the branches of the bifurcation diagram in the area of periodic solutions assumes positive values, then we deal with an increase in the effective value of the voltage induced on the piezoelectric electrodes. For its negative values, a decrease in the RMS voltage is observed at the piezoelectric electrodes.
- With increasing overlapping of the cut halves of the potential barrier, in a wide range of variability of the dimensionless excitation frequency, a reduction in the efficiency of energy harvesting is observed, which is confirmed by the diagrams of RMS voltage values presented in the graphs (Figure 4). The limitation of energy-harvesting efficiency, caused by the increase in the value of parameter *d*, is determined by the reduction in the width measured between the potential barriers.

The presented graphs clearly indicate that when designing energy-harvesting systems, the impact of hysteresis caused by overlapping potential barriers should be avoided or minimized. From an engineering point of view, such characteristics of potential barriers can be found in mechanical systems that have been pre-deformed, or in the case of shape-memory materials.

## Data Availability

Data is contained within the article.
